# The Effect of the Type of Screw Fixation Used in the Treatment of Slipped Capital Femoral Epiphysis

**DOI:** 10.1155/2022/9143601

**Published:** 2022-09-20

**Authors:** Tyler Rudolph, Katie Rooks, Haemish Crawford, Michael van der Merwe

**Affiliations:** Starship Childrens' Hospital, Auckland District Health Board, Auckland, New Zealand

## Abstract

**Background:**

Slipped capital femoral epiphysis (SCFE) remains among the most common hip disorders in the adolescent population. The management of SCFE remains controversial; however, the aim of fixation is to stabilize the physis and prevent further slippage. In situ fixation remains the gold standard; however, in the young population, it can lead to reduced femoral neck growth and complications such as leg length discrepancies. The ideal form of in situ fixation for mild to moderate SCFE would stabilize the slip and allow continued proximal femoral growth. This study aimed to determine if partially threaded screws allowed more neck growth than fully threaded screws.

**Methods:**

A retrospective review of the radiographs of all patients undergoing in situ fixation for SCFE using partially threaded and fully threaded screws. Measurements included neck length, neck-to-screw ratio, neck shaft angle, neck width, and articular-trochanteric distance. Parameters were compared over a two-year period to determine whether there was any difference in proximal femoral growth between the two types of screws.

**Results:**

Fully threaded screw neck length increased by 5 mm versus 5 mm for proximally threaded screws (*P* ≤ 0.001). No significant difference was observed between the two groups with respect to neck width, neck shaft angle, and articular-trochanteric distance.

**Conclusions:**

No difference was observed in proximal femoral growth. Regardless of which type of fixation is used, neck length continues to increase by approximately 3 mm per year.

## 1. Introduction

Slipped capital femoral epiphysis (SCFE) remains one of the most common hip disorders in adolescents with an increased incidence in New Zealand of 34 per 100,000 [1]. Occurring mostly in those aged 9–16 [2–4], SCFE predominantly affects males over females [3]. Treatment remains controversial for acute or severe SCFE; however, the gold standard of treatment for mild to moderate SCFE remains in situ fixation [5–7].

The aim of in situ fixation is to arrest physeal growth and prevent further slip of the physis [4, 8]. However, in young children with significant growth remaining, early physeal closure can lead to complications including leg length discrepancy [9], coxa vara, and femoroacetabular impingement [10–17]. The outcome of these complications for children can mean pain, deformity, and arthritis [9, 13, 14, 18, 19]. As a result, attempts have been made to reduce these complications using Kirschner wire (K-wire) fixation. Seller et al. [20] showed there were complications with K-wire fixation such as migration in the bone requiring further operations.

In the younger child, the ideal form of fixation would allow for continued proximal femoral growth while preventing further slippage, thus reducing complications of decreased proximal femoral growth, yet ensuring stabilization of the SCFE [21]. New modifications of proximal femoral fixation are being released to address these concerns including the Hansson hook-pin (Swemac) and free gliding screws (Pega Medical) [1]. Currently, at our institution and worldwide, the most common mode of fixation remains a standard cannulated screw [6, 7]. Both fully threaded and partially threaded options are available, and it is unclear in the literature whether partially threaded allow for more femoral neck growth.

The primary aim of this study was to determine which form of fixation (partially threaded or fully threaded cannulated screws) allowed for continued femoral neck growth (as determined by femoral neck length) over time.

Secondary objectives were to look at other aspects of femoral neck growth, including femoral neck width, articular-trochanteric distance, and neck shaft angle.

## 2. Materials and Methods

Ethics approval was obtained from the Auckland District Health Board Research Committee. All patients aged 5–15 years undergoing fixation of slipped capital femoral epiphysis at a national children's hospital from January 1, 2004, to December 31, 2014, were retrospectively identified from electronic records. A retrospective analysis of all radiographs was then performed. Inclusion criteria include fixation for any grade slip with partially threaded and fully threaded cannulated hip screws, unilateral or bilateral SCFE, a minimum of two years of follow-up with satisfactory radiographs, and l time point to allow a change in neck growth to be detected. Exclusion criteria include patients treated with an open reduction or modified Dunn osteotomy and fixation other than fully or partially threaded screws and inadequate follow-ups.

All radiographic measurements were performed by three blinded, skilled observers: an orthopedic surgeon and two orthopedic surgery residents (fellows). Any discrepancies in the measurements were evaluated the second time by all three observers and the average of the results was determined.

### 2.1. Radiographic Measurements

Parameters measured on radiographs included femoral neck length, neck length to screw ratio, femoral neck width, articular-trochanteric distance, and neck shaft angle.

**Figure 1:**
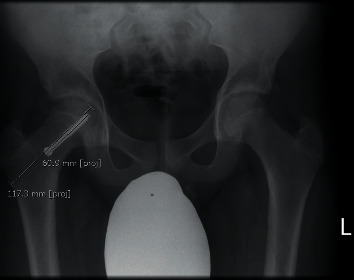
Neck length to screw length ratio measurement. Length of the screw is first measured, and a second measurement in line with the screw extending from the lateral cortex to the articular surface is then made. This gives a ratio that is used for comparative views. This measurement is used to determine proximal femoral neck growth over time. This method of measuring proximal femoral growth accounts for rotation between films.

Femoral neck length was determined by measuring a line drawn from the lateral cortex of the femur, in line with the superior boarder of the screw to the apex of the femoral head (Figure 1). The neck length to screw ratio was then measured to reduce any measurement error produced by rotation of the femur between films. This gave us an accurate scale in which to compare the growth to. This measurement tool was based on a study by Wölfle–Roos et al. [22].

**Figure 2:**
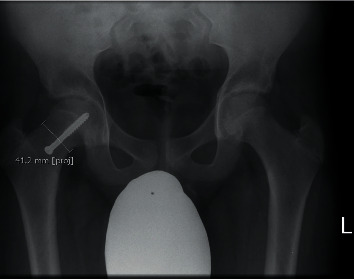
Neck width measurement. Neck width is measured at the narrowest point of the neck. This measurement is used to assess proximal femoral growth.

Femoral neck width was determined by identifying the narrowest point of the femoral neck and measuring the width at an angle perpendicular to the neck shaft axis as described by Sailhan et al. [23] (Figure 2).

**Figure 3:**
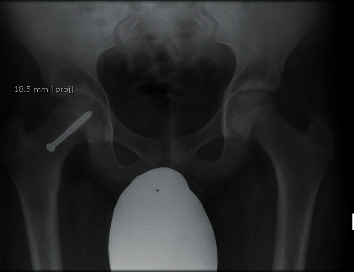
Articular-trochanteric distance. A horizontal line is drawn from the top of the femoral head and extending laterally. The distance between the tip of the greater trochanter and this line is then measured. This measurement is used to assess for trochanteric overgrowth.

Articular-trochanteric distance (Figure 3) was obtained by measuring a line from the tip of the greater tuberosity of the femur perpendicular to a line crossing both superior articular surfaces as described by Sailhan et al. [23].

**Figure 4:**
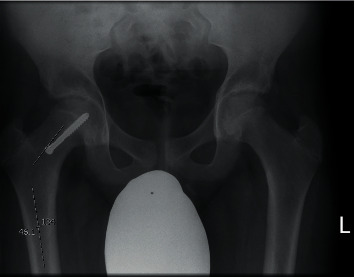
Neck shaft angle. A longitudinal line is drawn down the centre of the femoral neck. The angle between a longitudinal line down the shaft of the femoral neck and the shaft of the femur is measured. This angle represents the neck shaft angle.

Neck shaft angle was obtained by measuring the angle between the femoral neck axis and the femoral shaft axis as described by Sailhan et al. [23] (Figure 4).

## 3. Results

### 3.1. Demographic Data

From January 1, 2004, to December 31, 2014, a total of 199 individuals were identified as having undergone in situ fixation for slipped capital femoral epiphysis. Of these individuals, 70 had bilateral fixation at the time of surgery or within the 2-year follow-up period. Of these 199 individuals, 108 had suitable radiographic follow-ups for further analysis. The mean age was 11.5 (range 7–15), with men accounting for 54.8% and women 45.2%. Pacific Islanders made up the majority of patients, accounting for 51.8%. The remainder of the cohort was made up as follows: Maori 26.1%, New Zealand European 17.1%, Asian 2.5%, and others 2.1%. Further breakdown by screw type is depicted in Table 1. No significant differences were observed with respect to age, gender, and ethnicity between the two groups. Mean Southwick angle for partially threaded screws is 35.2° and fully threaded 30.5°.

### 3.2. Measurements

Neck length (Table 2), regardless of fixation type, was shown to increase by 7 mm over a two-year period (*P*-value <0.001, *N* = 107). Neck length was determined by measuring along the orientation of the screw. The subanalysis determined that the fully threaded screws (*N* = 25) increased by 7 mm and the partially threaded screws (*N* = 82) increased by 7 mm over two years (*P* value 0.745).

The neck-to-screw ratio (Table 3) (*N* = 105) was shown to increase by 8% (*P* value <0.001) over a two-year period in the combined group. When compared, the fully threaded screws (*N* = 25) increased by 6.6% and the partially threaded screws by 8% (*N* = 80, *P* value 0.19).

Neck width (Table 4) (*N* = 109) was shown to increase by 5 mm over 2 years in the overall group (*P* value <0.001). In comparison, fully threaded screws (*N* = 25) increased by 5 mm and partially threaded screws (*N* = 84) by 5 mm (*P* value 0.837).

Neck shaft angle (Table 4) was shown to decrease by 2° over a two-year period in the overall group (*N* = 108) (*P* value 0.332). In comparison, fully threaded screws (*N* = 26) decreased by 2° and partially threaded screws (*N* = 82) by 1° (*P* value 0.914).

Articular-trochanteric distance (Table 4) in fully threaded screws (*N* = 25) decreased by 4 mm and in partially threaded screws (*N* = 82) by 4.2 mm (*P* value 0.017).

## 4. Discussion

The results of this study demonstrate that there is no significant difference in femoral neck length between fully threaded cannulated screws and partially threaded cannulated screws. With respect to other parameters of proximal femoral neck growth, there is no significant difference between the two groups. Clinically, this means that either screw can be used on the young child depending on the surgeon's preference.

The consensus is that the treatment of SCFE in the younger child should aim to stabilize the physis and prevent further slippage, while allowing for continued neck growth [24]. Therefore, several fixation types have been used, such as Kirschner wires, Hansson hook-pin, proximally threaded screws, and more recently the free gliding screw. In a study by Wölfe–Roos et al. [22], they compared the use of Kirschner wire (K-wire) versus screw fixation. They used a similar measurement technique by comparing the growth of the proximal femur using the screw-to-neck length ratio. In their study, they found that screw fixation had significantly lower femoral neck growth compared to K-wire fixation (8.9% ± 5.7%). Örtegren et al. [24] performed a retrospective review of patients treated with the Hansson hook-pin over an 8 year period. They found the affected hip grew by approximately 7.1 mm by the time of physeal closure (mean interval, 34 m). Sailhan et al. [23] conducted a study whereby they compared the femoral neck growth of the affected side treated with a proximally threaded screw to the unaffected side. They found proximally threaded screws allowed for an average of 5 mm of neck length growth over an average period of 31 months; they concluded proximally threaded screws allow for continued growth after fixation and now routinely use this type of fixation. More recently, the free gliding screw has been developed which theoretically allows more femoral neck growth due to the telescoping effect. A study by Leblanc et al. [25] compared the use of the free gliding screw to a static threaded screw. They concluded this implant is at least not inferior to static fixation with regards to maintaining proximal femoral growth and may prove a viable alternative in the future with further research.

Although SCFE management in the younger child remains controversial, the results of our study suggest that despite the type of fixation used, femoral neck growth will continue. Kirschner wires, Hansson hook-pins, and proximally threaded wires have all been shown to allow continued neck growth. Our study supports the notion that regardless of fixation type (partially thread or fully threaded), growth will continue. These findings were similar to those of Breaud et al. [26] who found that in their study, hip growth continued after cannulated fixation of hips. Therefore, the decision on which implant to use should not be made on the grounds of maintaining proximal femoral growth, but on other issues of in situ fixation such as further slippage, difficulty with the removal of metal ware, and implant failure, an area this paper does not aim to address.

The limitations of this study include the inaccuracy associated with measuring two-dimensional x-rays. Without CT-guided measurement, the true measurement of femoral neck length cannot be obtained. To reduce the inaccuracy of X-ray measurements, the screw-to-neck length ratio was used to show the neck length measurements we observed were indeed real. Although CT is the gold standard, in practice, this is not viable for research purposes owing to the risks associated with radiation exposure. This study showed femoral neck growth over a period of two years; however, some children will continue to grow beyond this, and therefore, the true impact of femoral neck length would be best observed with longer-term follow-up. It also must be noted that there has been a greater number of partially threaded screws used in the last 10 years, as the transition to fully threaded screws has been recent. In our study, we did not explore the association between initial Southwick angle and degree of growth after in situ fixation; however, both groups had a similar mean Southwick angle. Finally, our study included hips that underwent prophylactic fixation; therefore, it is possible that the growth of an unaffected hip may be different to that of the affected hip.

We were able to demonstrate the articular-trochanteric distance decreased over the two-year period; and the –neck shaft angle was relatively unchanged. This is suggestive of the fact that the articular-trochanteric distance decreases because of trochanteric overgrowth relative to the femoral neck, as opposed to coxa vara malformation.

## 5. Conclusions

This study found no difference between partially threaded screws and fully threaded screws; therefore, either remains a viable option when considering in situ fixation. We believe that in situ fixation remains a suitable option for younger children with SCFE and is a procedure that a general orthopedic surgeon should be able to perform. Further research on this topic is needed to clearly demonstrate the natural history of femoral neck growth following in situ fixation using the different methods available. The use of CT scanning would allow the most accurate assessment and would help in achieving consensus on the best method of fixation for SCFE in the younger child. A longer-term follow-up would allow a more accurate analysis of femoral neck growth over time, particularly in the younger child.

## Figures and Tables

**Table 1:** Demographic information on patient cohort.

	Fully threaded	Partially threaded	*P* value
Patient age (years)	11.4	11.5	0.583
Male gender	57.4%	53.4%	0.598
Ethnicity			0.903
NZ European	17.6%	16.8%	
Māori	27.9%	25.2%	
Pacific Islander	51.5%	51.9%	
Other	1.5%	3.1%	
Asian	1.5%	3.1%	

**Table 2:** Neck length.

	6 weeks (mm)	2 years (mm)	Observed difference
Fully threaded	101	108	7 mm
Partially threaded	103	110	7 mm
			*P* value 0.745

**Table 3:** Neck-to-screw ratio.

	6 weeks	2 years	Observed difference (%)
Fully threaded	1.178	1.256	6.6%
Partially threaded	1.402	1.541	8%
			*P* value 0.190

**Table 4:** Neck width, neck shaft angle, and articular-trochanteric distance.

	6 weeks	2 years	Observed difference (mm)
Neck width			
Fully threaded	36.2	41.1	4.9
Partially threaded	37.9	43.3	5.4
			*P* value 0.837
Neck shaft angle			
Fully threaded	138.7	136.5	2.2 (degrees)
Partially threaded	137.9	136.7	1.2
			*P* value 0.914
Articular-trochanteric distance			
Fully threaded	26.3	22.3	4
Partially threaded	22.9	18.7	4.2
			*P* value 0.017

## Data Availability

The data are not publicly available and have been kept on a secure server as per ethics guidelines. The data used to support this study are available from the corresponding author upon request.
